# Successful treatment of refractory palmoplantar pustulosis by upadacitinib: report of 28 patients

**DOI:** 10.3389/fmed.2024.1476793

**Published:** 2024-11-06

**Authors:** Na Du, Jingyi Yang, Yiwen Zhang, Xinyan Lv, Lei Cao, Wei Min

**Affiliations:** ^1^Department of Dermatology, The First Affiliated Hospital of Soochow University, Suzhou, China; ^2^Jiangsu Institute of Clinical Immunology, The First Affiliated Hospital of Soochow University, Suzhou, Jiangsu, China; ^3^Jiangsu Key Laboratory of Gastrointestinal Tumor Immunology, The First Affiliated Hospital of Soochow University, Suzhou, Jiangsu, China; ^4^Jiangsu Key Laboratory of Clinical Immunology, Soochow University, Suzhou, Jiangsu, China

**Keywords:** refractory, palmoplantar pustulosis, Janus kinase 1-selective inhabitor, upadacitinib, clinical efficacy and safety

## Abstract

**Background:**

Upadacitinib, a specific JAK1 inhibitor, has minimal effect on other JAK subtypes. It influences the inflammatory process in various ways. Upadacitinib has been approved for conditions such as rheumatoid arthritis, psoriatic arthritis, atopic dermatitis, and ulcerative colitis in various countries. The purpose of this study is to assess the clinical efficacy and safety of upadacitinib in patients with refractory palmoplantar pustulosis who have not responded to conventional treatments (e.g., Acitretin, Tripterygium wilfordii Hook F, cyclosporine, methotrexate).

**Methods:**

We conducted a retrospective collection of clinical data from 28 patients who received upadacitinib treatment at the Department of Dermatology, First Affiliated Hospital of Suzhou University, from July 2022 to December 2023. We evaluated the Palmoplantar Pustulosis Area and Severity Index (PPPASI) scores, Dermatology Life Quality Index (DLQI) scores, and Physician’s Global Assessment (PGA) scores before and after treatment. We also recorded any adverse events during the treatment process.

**Results:**

A total of 28 patients were diagnosed with PPP, including 10 males and 18 females, and 8 patients (3 males and 5 females) were diagnosed with SAPHO syndrome. The mean age was (36.3 ± 10.5) years. After 12 weeks of treatment, PPPASI scores decreased from baseline (13.86 ± 2.76) to (5.56 ± 1.08), with a statistically significant difference (*p* < 0.05). DLQI scores decreased from (12.55 ± 4.56) to (2.03 ± 1.13), also showing a statistically significant difference (*p* < 0.05). Additionally, 20 patients achieved a PGA score of 0/1. No severe adverse events were reported during the treatment and follow-up period.

**Conclusion:**

Upadacitinib could be an additional safe and effective treatment for treating refractory palmoplantar pustulosis with a potential benefit on patients’ quality of life. Further studies are needed to assess its short-and long-term efficacy and safety in larger sample sizes in randomized double-blind controlled trials.

## 1 Introduction

Palmoplantar pustulosis (PPP) is a chronic and relapsing inflammatory condition with phases of erythema, scaling, and aseptic pustules on the palms and soles. Palmoplantar pustulosis primarily affects the palms and soles of the feet, causing discomfort and loss of function, significantly reducing the patient’s quality of life. These symptoms significantly hinder the patients’ daily lives (1, 2). Additionally, there are limited treatment options available for PPP, and definite pharmaceutical interventions have yet to be identified. In field of medicine, Janus kinase (JAK) inhibitors are being increasingly used. However, there is still limited understanding of the clinical efficacy and safety of JAK inhibitors in treating PPP. Therefore, the main objective of this study was to analyze the clinical use of JAK inhibitors in treating PPP.

## 2 Materials and methods

### 2.1 Study subjects

For this study, clinical data of patients diagnosed with palmoplantar pustulosis (PPP) who underwent upadacitinib treatment at the Department of Dermatology, First Affiliated Hospital of Suzhou University, between May 2022 and August 2023 were retrospectively analyzed. The inclusion criteria were as follows: ① All patients conformed to the diagnostic criteria for PPP (3), characterized by histopathological features including intraepidermal spongiform pustules, infiltration of inflammatory cells such as neutrophils and monocytes within pustules, and presence of inflammatory cell infiltration within the dermis; ② Patients who had previously attempted systemic treatment and/or phototherapy but had not achieved effective results or could not tolerate them. The exclusion criteria were as follows: ① Patients concurrently receiving other systemic treatments; ② Women who were pregnant, breastfeeding, or planning to conceive during the medication period; ③ Patients with severe cardiovascular, cerebrovascular diseases, severe hepatic or renal dysfunction, or existing neoplastic conditions; ④ Patients unable to adhere to the prescribed medication regimen or complete the follow-up. Patients who fulfilled the inclusion criteria were included in the study and the analysis. This study has received approval from the Ethics Committee of Drug Clinical Trials of the First Affiliated Hospital of Suzhou University (Audit number: By the Ethics Committee Research Approval # 315), and all patients have provided informed consent.

### 2.2 Research methods

#### 2.2.1 Clinical data

Among the 28 PPP patients, there were 10 males and 18 females, with an average age of (36.3 ± 10.5) years and disease duration ranging from 5 months to 6 years. A biopsy was performed on all patients and confirmed the diagnosis of PPP. The histopathological examination of the patient’s skin lesions revealed the presence of spongy pustules within the epidermis, accompanied by inflammatory cell infiltration comprising neutrophils and monocytes within the pustules, as well as inflammatory cell infiltration within the dermis. Among them, 2 had a history of smoking (1 case for 1 year, 1 case for 3.2 years), while the rest denied any smoking history. Past medical history revealed that 15 patients (53.6%) had received treatment with Acitretin, of which 11 showed a poor response and discontinued the medication, and 4 developed adverse reactions and stopped the treatment; 6 patients (21.4%)were treated with Tripterygium wilfordii Hook F (TwHF), With 4 showing inadequate efficacy and 2 discontinuing due to adverse reactions; 5 patients (17.9%) were treated with cyclosporine, of which 2 had poor response, 1 showed short-term improvement but relapsed after discontinuation; 6 patients (21.4%) received methotrexate, all with poor efficacy; 6 patients (21.4%) were treated with adalimumab, 2 showed short-term improvement but lost efficacy over the long term, 1 had poor response; 5 patients (17.9%)were treated with secukinumab, of which 3 showed significant short-term improvement, 1 had poor long-term response, and 1 had poor response.

#### 2.2.2 Treatment method

During the treatment process, according to the instructions for upadacitinib, patients orally received a daily dose of 15 mg.

#### 2.2.3 Efficacy evaluation and criteria

We primarily evaluated patients’ efficacy using the following indicators and judged based on relevant criteria: Palmoplantar Pustulosis Area and Severity Index (PPPASI) scores (4): We scored patients’ erythema, pustules, scales, and affected area on the palms and soles, while calculating the PPPASI improvement rate. The formula for calculating the PPPASI improvement rate is (pre-treatment score - post-treatment score)/pre-treatment score × 100%. We defined PPPASI improvements of 50, 75, and 90% as PPPASI50, PPPASI75, and PPPASI90 (5), respectively. Dermatology Life Quality Index (DLQI) (5): This index is primarily used to assess the impact of skin disease on patients’ quality of life over the past week. It comprises 10 questions covering various aspects, and responses of varying degrees reflect the extent of impact. Total scores range from 0 to 30, with higher scores indicating a greater impact on quality of life. Physician’s Global Assessment (PGA) (6, 7): Based on the overall skin condition of patients, we categorized them on a scale of 0–5, where 0 indicates no lesions and 5 indicates severe lesions.

#### 2.2.4 Statistical analysis

Data analysis was conducted using SPSS 23.0 statistical software. For continuous variables, mean ± standard deviation (x ± s) was used for representation. Since the data was normally distributed, *t*-tests were used to compare differences between the two sets of data. Categorical data were expressed as frequencies (percentages). Differences were considered statistically significant when the *p*-value was less than 0.05.

## 3 Results

### 3.1 Clinical efficacy evaluation

**Figure 1:**
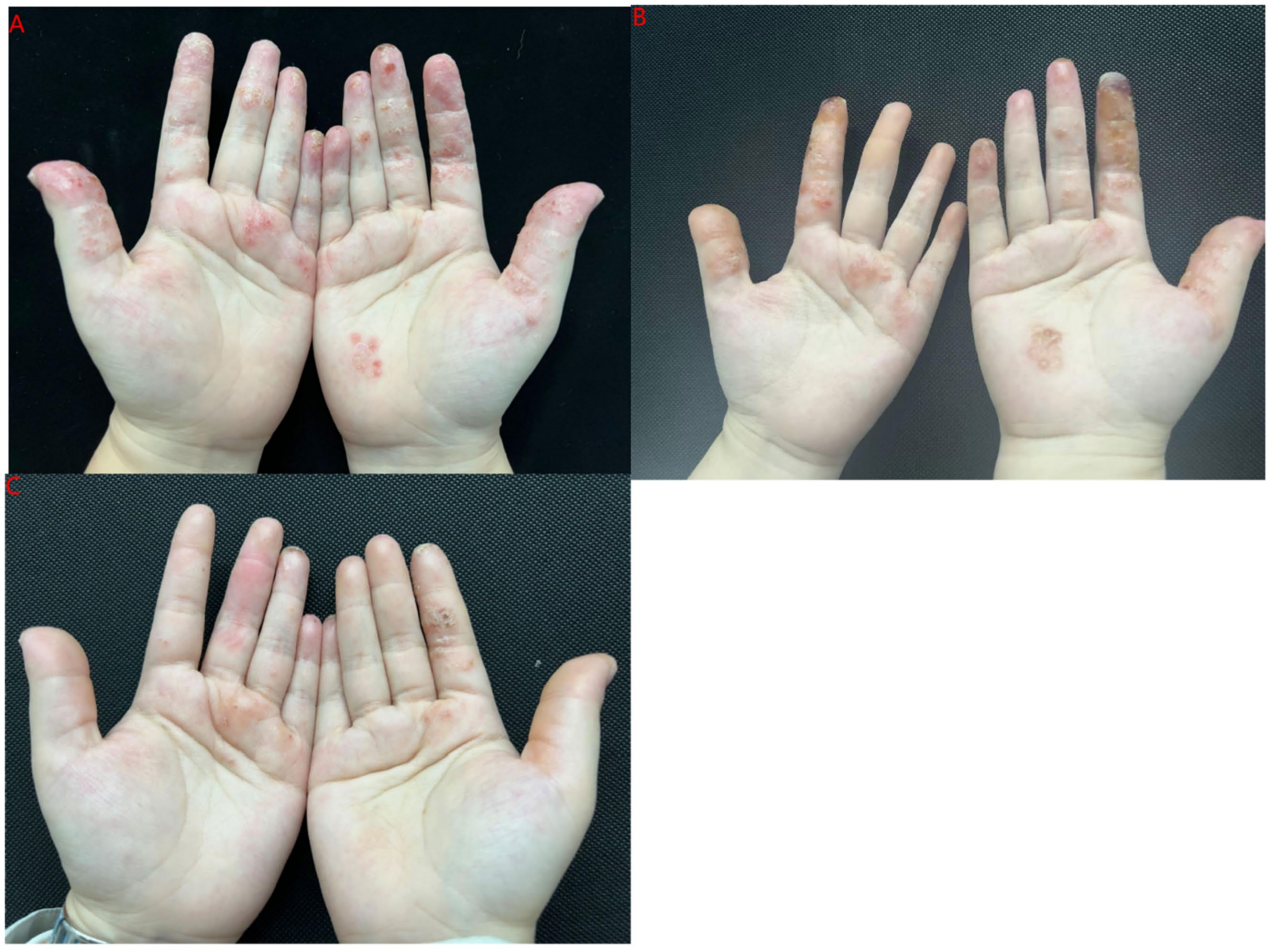
(A) Before upadacitinib treatment (Case 5); (B) 2 weeks upadacitinib treatment (case 5); (C) 12 weeks upadacitinib treatment (case 5).

Over a treatment period of 12 weeks, the 28 PPP patients completed the treatment. The results revealed that the PPPASI score decreased from a pre-treatment value of (13.86 ± 2.76) to (5.56 ± 1.08) after 12 weeks of treatment. The reduction in PPPASI score post-treatment was statistically significant (*t* = 2.418, *p* < 0.001). Notably, among them, 25 patients achieved PPPASI50, 20 patients achieved PPPASI75, and 18 patients achieved PPPASI90. Additionally, 20 patients achieved a PGA score of 0/1, accounting for 71.4% of the total (Table 1). At 12-week follow up 28 patients achieved control of their disease with no reported cases of flares (Figure 1).

**Table 1:** Comparing the different treatment time to achieve PPPASI50/PPPASI75/PPPASI90 cases of patients.

Time of therapy	PPPASI50 (%)	PPPASI75 (%)	PPPASI90 (%)
4w	18 (64.3)	8 (28.6)	3 (10.7)
8w	20 (71.4)	15 (53.6)	11 (39.3)
12w	25 (89.3)	20 (71.4)	18 (64.3)

### 3.2 Patient self-assessment scores

Among the 28 PPP patients, all of them showed an improvement in DLQI scores. The DLQI score decreased from a pre-treatment value of (12.55 ± 4.56) to (2.03 ± 1.13) after 12 weeks of treatment. The difference in DLQI scores before and after treatment was statistically significant (*p* < 0.05) (Table 2).

**Table 2:** Comparison of DLQI scores and number of patients with PGA0/1 before and after treatment in PPP patients.

Time of therapy	DLQI	*t*	*p*	PGA (0/1, %)
0w	12.55 ± 4.56	9.498	<0.001	0
4w	7.98 ± 2.45	16.083	<0.001	4 (14.3)
8w	4.49 ± 2.28	21.463	<0.001	12 (42.9)
12w	2.03 ± 1.13	19.968	<0.001	20 (71.4)

### 3.3 Safety assessment

During upadacitinib treatment, none of the 28 patients experienced severe adverse events. Among them, 4 patients developed acneiform rash. However, these lesions gradually improved after using topical adapalene gel and fusidic acid cream for 2 weeks. Bone and joint pain improved in 8 patients with SAPHO syndrome after treatment. The visual analog scale (VAS) score was 4–5, and 0–1 after treatment. The PPPASI score decreased from a pre-treatment value of (11.76 ± 3.66) to (7.56 ± 2.08) after 12 weeks of treatment. However, two patients showed less improvement in bone and joint pain and skin lesions compared to the other six patients. Moreover, two patients experienced transient transaminitis. Both had normal liver function tests before treatment and had no history of related treatments. The extent of elevation did not exceed twice the normal upper limit. In two patients, liver enzymes gradually returned to normal levels after they took Polyene phosphatidylcholine orally. In addition, one of the patients showed a slight increase in creatinine levels. After excluding organic lesions and other potential causes, no specific intervention was needed. The renal function was rechecked 3 weeks later and had returned to normal.

## 4 Discussion

As a rare chronic inflammatory and relapsing disease, palmoplantar pustulosis (PPP) has an incidence ranging from 0.05 to 0.12% (8). There is ongoing debate regarding whether psoriasis and PPP should be considered as the same disease or distinct variants. However, the results of several studies suggest that PPP can be considered as a separate entity. Studies have shown that females and smoking are risk factors for PPP (9), which is in line with the present study, where 18 out of 28 patients were females, which is consistent with the literature, and two patients had a history of smoking. Other factors that may contribute to or worsen PPP include infections (such as tonsillitis and dental infections), psychological stress, and certain medications like TNF and IL-17 inhibitors, which could also trigger PPP (10–12).

The pathophysiological mechanisms of PPP are not fully understood at present, and only a minority of PPP patients have known genes associated with pustular psoriasis. Among European patients, mutations in CARD genes are linked to PPP, as well as mutations in the AP1S3 gene (13). Research has shown that certain natural or externally applied substances, such as human cathelicidin antimicrobial peptide 18 (hCAP-18)/LL-37, may have a role in the initial development of PPP. These antimicrobial peptides come from the secretions of the end parts of sweat ducts in eccrine sweat glands and could contribute to triggering inflammation, affecting the production of factors that cause inflammation, and maintaining the balance between Th17 and Treg cells (14). Meanwhile, TLN-58, derived from hCAP-18, is also expressed in PPP lesions, promoting neutrophil recruitment and pro-inflammatory cytokine production, thus contributing to inflammation. Research has also indicated that the gene encoding the inflammatory cytokine IL-36γ is upregulated in PPP patients, suggesting the involvement of the IL-36 pathway disruption in the development of skin lesions (15). Activation of IL-36 receptors leads to the activation of nuclear factor (NF)-κB. This, in turn, induces the secretion of IL-8, pro-inflammatory cytokines TNF-*α*, IL-1, and IL-23, as well as enhances Th-17 activity. IL-8 can chemotactically attract neutrophils, acting as a catalyst for aseptic pustules. In addition to the hyperactivity of the IL-36 pathway, PPP patients also exhibit activation of the IL-17 pathway, with significantly elevated levels of certain cytokines from the IL-17 family (such as IL-17A, IL-17C, IL-17F) in skin tissues (16).

Due to the complex pathogenesis, approved standardized treatments are lacking. Clinically, the treatment of PPP involves a range of approaches, including the use of topical medications such as corticosteroids, retinoids, and keratolytics. However, these treatments often have limited efficacy, and systemic therapy or phototherapy is often necessary. Preferred systemic agents typically include acitretin, methotrexate, TwHF, and cyclosporine, among others (17). Tripterygium wilfordii Hook F (TwHF), a member of the Celastraceae family of vine-like plants, is an important drug in traditional Chinese medicine, first recorded in the 16th-century Compendium of Materia Medica. TwHF has significant anti-inflammatory and immune-modulating properties and is widely used in various autoimmune-mediated inflammatory diseases. Notably, the compounds celastrol and triptolide derived from TwHF exhibit efficacy against conditions characterized by inflammation, such as RA (18). Even though TwHF and its extracts have been proven effective in treating inflammatory and immune-related disorders, their potential side effects should be carefully considered. In the UK, consumers have been advised not to use unlicensed herbal products containing lei gong teng (tripterygium wilfordii, also known as Thunder God Vine or Seven-step vine) due to concerns about serious side effects on fertility, liver, kidneys, immune system, blood, and heart (19). Han et al. reported that gastrointestinal complaints, aberrant hepatocytes, and reproductive dysfunction are the most common side effects of TwHF extracts (20). A meta-analysis of 14 studies revealed TwHF-related toxicity in systemic application, including menstrual disorders in women, dry mouth, gastrointestinal complaints, swelling of the lower limbs, abnormal hepatocytes, and abnormal routine blood results (21). Another meta-analysis reveals that TwHF may also cause higher reproductive toxicity, severe skin responses, hematologic problems, and cardiovascular events (22). So before initiating treatment with TwHF, we conducted thorough communication with the patients and employed it upon obtaining their informed consent. Moreover, during the medication process, we regularly monitor blood routine, as well as liver and kidney functions. Nonetheless, some patients discontinue treatment due to slow response, inadequate efficacy, or severe adverse reactions. Regardless of the treatment method used, relapses after discontinuation remain a common challenge.

Janus kinases (JAKs) are a class of intracellular non-receptor tyrosine kinases consisting of subtypes JAK1, JAK2, JAK3, and TYK2. They play a role in the downstream signaling of various type I and type II cytokine receptors, such as IL-6, IL-17, IL-19, IL-20, IL-22, IL-23, and IFN-*γ*. These cytokines and pathways are involved in immune cell differentiation, maturation, humoral immune regulation, immune barrier function, cell proliferation, and apoptosis, and they play crucial roles in autoimmune, allergic, and inflammatory reactions (23, 24). Upadacitinib, a specific JAK1 inhibitor, has minimal effect on other JAK subtypes. This minimizes its interference with the normal functions of other JAK subunits. Further studies show that upadacitinib affects the growth and activation of inflammatory cells like CD4+ T cells, neutrophils, and dendritic cells. It also reduces the production of inflammatory cytokines such as IL-6, IL-17, IL-2, IL-36, and IFN-*γ*, which in turn affects the differentiation of Th cells and the infiltration of inflammatory cells. In summary, it influences the inflammatory process in various ways (25). Upadacitinib has been approved for conditions such as rheumatoid arthritis, psoriatic arthritis, atopic dermatitis, and ulcerative colitis in various countries. Numerous clinical trials and real-world data studies have confirmed its effectiveness and safety in treating inflammatory diseases like atopic dermatitis (26).

Currently, there is limited literature on the use of upadacitinib in PPP treatment, with few reports available (14, 27). In this study, oral upadacitinib was used to treat PPP patients. The results showed that short-term use of upadacitinib significantly improved clinical symptoms in PPP patients who had previously shown poor response, ineffectiveness, or intolerance to systemic treatment. The treatment was well-tolerated and also improved the quality of life for the patients. Prolonged use of upadacitinib may help maintain effectiveness and reduce disease relapse, potentially offering a new treatment option for PPP. We will continue treating 28 patients with stable disease with upadacitinib at a dose of 15 mg/day to achieve long-term control of the skin lesions. However, due to the limitations of this study, such as a relatively small sample size, short treatment and follow-up period, lack of a control group, and limited literature on upadacitinib’s use for PPP, more clinical research data is needed to assess its safety and effectiveness. This will also help determine the best approach for maintenance therapy.

## Data Availability

The original contributions presented in the study are included in the article/Supplementary material, further inquiries can be directed to the corresponding authors.
