# Supplementary material for: Successful treatment of refractory palmoplantar pustulosis by upadacitinib: report of 28 patients
Source: Front Med (Lausanne). 2024 Nov 6;11:1476793. doi: 10.3389/fmed.2024.1476793 (PMC11576273; doi:10.3389/fmed.2024.1476793)
Supplement: Supplementary file 1 [file Table_1.DOC]

Baseline demographic and clinical characteristics of 28 patients

| **Characteristic** |  |
| --- | --- |
| Age, mean±SD, years | 36.3±10.5 |
| Sex, n (%) | |
| Male | 10 (35.7) |
| Female | 18 (64.3) |
| Onset age of PPP, mean±SD, years | 37.73±11.16 |
| Duration of PPP, mean±SD, years | 4.05±3.08 |
| p-NRS^a^, mean±SD | 7.38±2.48 |
| s-NRS^b^, mean±SD | 5.63±3.59 |
| DLQI^c^, mean±SD | 12.55±4.56 |
| Baseline PGA^d^, n (%) | |
| PGA 3 | 14 (50.0) |
| PGA 4 | 14 (50.0) |
| Baseline PGA activity^e^, n (%) | |
| PGA activity 1 | 3 (10.7) |
| PGA activity 2 | 4 (14.3) |
| PGA activity 3 | 17 (60.7) |
| PGA activity 4 | 4 (14.3) |
| Family history of PPP, n (%) | 3 (1.07) |
| Systemic treatment received before upadacitinib, n (%) | |
| Acitretin | 15 (53.6) |
| Tripterygium wilfordii Hook F | 6 (21.4) |
| Cyclosporine | 5 (17.9) |
| Methotrexate | 6 (21.4) |
| Adalimumab | 6 (21.4) |
| Secukinumab | 5 (17.9) |

p-NRS, pruritus numeric rating scale; s-NRS, sleeplessness numeric rating scale; PGA, Physician's Global Assessment ; DLQI, Dermatology Life Quality Index;

^a^Numeric rating scale scores ranged from 0 to 10, with higher scores reflecting worse itch.

^b^Numeric rating scale scores ranged from 0 to 10, with higher scores reflecting worse sleeplessness.

^c^DLQI scores ranged from 0 to 30, with higher scores reflecting worse influence on the quality of life.

^d^PGA scores classified the stage as clear (0), almost clear (1), mild (2), moderate (3), severe (4) ,very severe(5)according to the numbers of lesions.

^e^PGAactivity scores classified the activity as clear (0), almost clear (1), mild (2), moderate (3), severe (4), very severe(5)according to the rate of lesions with excoriations or crusts.
